# Survival benefit of primary tumor resection for gastric cancer with liver metastasis: A propensity score-matched, population-based study

**DOI:** 10.3389/fonc.2022.1039086

**Published:** 2022-11-17

**Authors:** Jiayan Wu, Jiandong Yu, Zhiping Chen, Hongquan Zhu, Chengrui Zhong, Yongling Liang, Ziyan Mai, Zejin Lin, Yunle Wan, Guolin Li

**Affiliations:** ^1^ Department of Hepatobiliary, Pancreatic and Splenic Surgery, The Sixth Affiliated Hospital, Sun Yat-sen University, Guangzhou, China; ^2^ Department of General Surgery, Jiangmen Central Hospital, Jiangmen, China

**Keywords:** gastric cancer, liver metastasis, primary tumor resection, prognosis, SEER

## Abstract

**Objectives:**

Gastric cancer with liver metastasis (GCLM) is highly aggressive and has a poor prognosis. This study aims to evaluate the survival benefit of primary tumor resection (PTR) for gastric cancer with liver metastasis.

**Methods:**

Data on patients with GCLM was extracted from the Surveillance, Epidemiology, and End Results (SEER) database from 2010 to 2015. A 1:1 propensity score matching (PSM) analysis was performed to minimize the heterogeneity between the PTR and no-PTR groups. The Kaplan–Meier method and Cox regression analysis were used to assess the impact of primary tumor resection (PTR) on overall survival (OS) and cause-specific survival (CSS).

**Results:**

A total of 3,001 patients with GCLM were included, with 328 patients treated with primary tumor resection (PTR), whereas the other 2,673 patients were not. Patients with PTR had a significantly higher OS and CSS rate than those without PTR in unmatched and PSM cohorts. In an unmatched cohort, the median OS was 12.0 months (95% CI, 10 months to 14 months) for those who underwent PTR and 4 months (95% CI, 4 months to 5 months) for those without PTR; the median CSS for those who underwent PTR was 12.0 months (95% CI, 10 months to14 months) and 4 months (95% CI, 4 months to 5 months) for those without PTR, respectively. After PMS, the median OS was 12.0 months (95% CI, 10 months to 17 months) for those who underwent PTR and 7 months (95% CI, 5 months to 10 months) for those without PTR, respectively; the median CSS for those who underwent PTR was 12.0 months (95% CI, 11 months to 17 months) and 7 months (95% CI, 5 months to 8 months) for those without PTR, respectively. In addition, multivariate Cox analysis in the PSM cohort showed that PTR, age, degree of tumor differentiation, and chemotherapy were independent prognostic factors for OS and CSS in GCLM. Specifically, PTR was a significant protective factor for OS (HR: 0.427; 95% CI, 0.325 to 0.561, P <0.001) and CSS (HR: 0.419; 95% CI, 0.313 to 0.561, P <0.001).

**Conclusion:**

Primary tumor resection improves the survival of gastric cancer patients with liver metastasis.

## Introduction

Gastric cancer is the fifth most common malignant tumor, and it is the fourth-leading cause of cancer-related deaths in the world. In 2020, over one million (1,089,103) new cases were reported, with an estimated 769,000 deaths worldwide (1). Besides, because of a lack of early symptoms, over 80% of gastric cancer patients were at an advanced stage at the time of diagnosis. Some of them had distant metastases. The metastases of gastric cancer include three routes: hematogenous, lymphatic, and peritoneal dissemination (2, 3); and the liver is the most common metastatic organ. For gastric cancer patients with liver metastasis (GCLM), guidelines regard palliative gastrectomy as a choice when surgery is unavoidable (4) and chemotherapy can be considered to be adopted (5, 6). The AIO-FLOT3 trial (7) showed that patients who were treated with gastrectomy and chemotherapy had better OS (22.9 vs. 10.7 months) than those treated with chemotherapy alone. However, the phase III study REGATTA trial (8) reported that the overall survival (OS) and progression-free survival (PFS) of patients who were treated with palliative surgery plus chemotherapy had no significant difference with those who were treated with chemotherapy only. Consequently, whether palliative gastrectomy improves the prognosis of GCLM remains unclear. The necessity of primary tumor resection for GCLM patients is a debatable issue.

Therefore, the aim of the study was to investigate the survival influence of primary tumor resection in GCLM patients based on the Surveillance, Epidemiology, and End Results (SEER) database.

## Materials and methods

### Ethics statement

This study was approved by the Ethics Committee of the Sixth Affiliated Hospital of Sun Yat-sen University, and this study does not need informed patient consent because the data was collected from the SEER database.

### Patients selection

**Figure 1:**
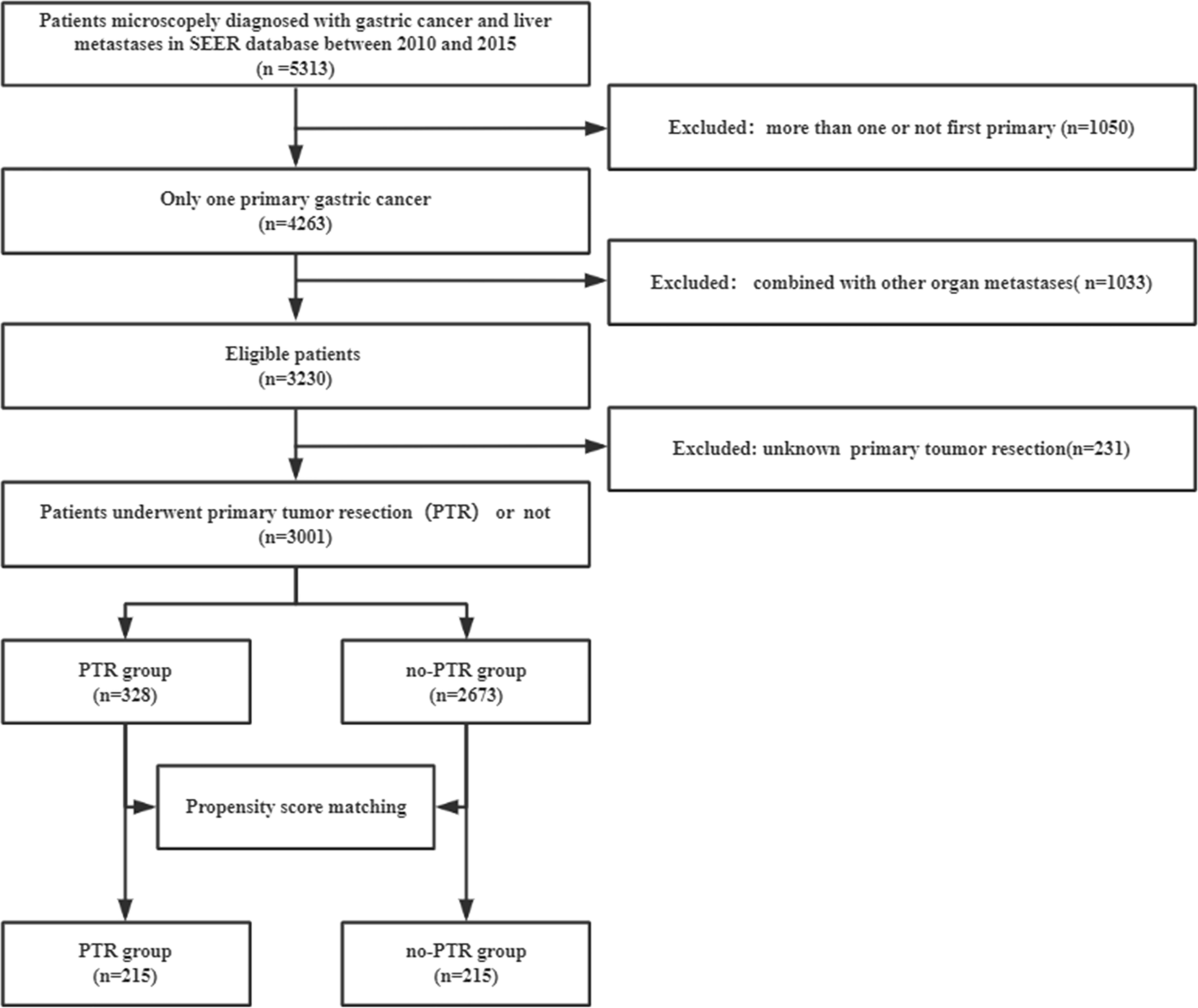
Flowchart of patient selection.

The Surveillance, Epidemiology, and End Results (SEER) program, sponsored by the National Carcinoma Institute of the United States of America and established in 1973, reported a variety of cancer cases with patient characteristics and patient survival from 19 regional areas of the USA, representing approximately 34.6% of the US population. We used the SEER*Stat software (version 8.4.0) with the ID number 10355-Nov2021 to obtain patient characteristics and survival information. This software was used to identify gastric cancer with liver metastasis (GCLM) (**Figure 1**). Firstly, GCLM patients were retrieved based on the Site and Morphology value ({Site and Morphology. Primary Site-labeled} = ‘C16.0,’ ‘C16.1,’ ‘C16.2,’ ‘C16.3,’ ‘C16.4,’ ‘C16.5,’ ‘C16.6,’ ‘16.7,’ ‘C16.8,’ ‘C16.9’). Secondly, gastric cancer with liver metastasis between 2010 and 2015 with a microscopic diagnosis was chosen. The inclusion criteria for our study were as follows: (1) being diagnosed with gastric cancer only; (2) confirmed liver metastases; (3) confirmed with histologically documented cancer; and (4) patients who had a primary tumor resection. The exclusion criteria in this study were as follows: (1) patients with more than one primary tumor or unknown cancer; (2) combined with other organ metastases; and (3) unknown primary tumor resection. Thirdly, all the variables collected in this study were as follows: (1) year of diagnosis (2010/2011/2012/2013/2014/2015); (2) age at diagnosis (>/<65y); (3) sex (Male/Female); (4) race (Black/White/Other); (5) marital status (Divorced/Married/Single/Other); (6) income (<60,000/>60,000); (7) residence (Metropolitan/Rural/Urban); (8) reporting source (Hospital/no-Hospital); (9) PRCDA (No/Yes); (10) original record (Hispanic/Non-Hispanic); (11) tumor differentiation (Grade I/Grade II/Grade III/Grade IV/Unknown); (12) tumor T-stage (T0/T1/T2/T3/T4/TX); (13) tumor N-stage (N0/N1/N2/N3/NX); (14) pathological type (adenocarcinoma/Gastrointestinal stromal/Intestinal type/Signet ring cell/Other); (15) tumor location (Body/Cardia/Fundus/Gastric antrum/Greater/Lesser/Pylorus/Other); (16) tumor size (<1 cm/>1 cm/Unknown); (17) chemotherapy (No/Yes); (18) radiotherapy (No/Yes); (19) primary tumor resection (No/Yes); and (20) survival rate.

### Propensity score-matching

The aim of the study was to research the effect of primary tumor resection in patients with gastric cancer with liver metastasis, and this research was a retrospective study. Consequently, the potential covariates between primary tumor resection and the non-surgery cohort were not balanced, which might distort the fundamental relationship of primary tumor resection with OS and CSS. Therefore, propensity score matching (PSM) was further performed to reduce this influence. And according to the nearest neighbor matching method with a setting caliper value of 0.1 (9), patients were propensity matched 1:1 into PTR and no-PTR groups. All the covariates used for matching in this study were as follows: age, year of diagnosis, sex, race, marital status, income, residence, reporting source, PRCDA, original record, tumor differentiation, tumor T-stage, tumor N-stage, pathological type, tumor location, tumor size, chemotherapy, and radiotherapy.

### Statistical analysis

The Kaplan‐Meier method was used to estimate OS and CSS before and after PSM, and the log-rank tests were conducted to compare survival differences in the PTR and no-PTR groups. The Cox proportional hazards regression method was performed to identify prognostic factors for OS and CSS. In the univariate Cox model, variables with a P-value <0.05 were further incorporated into the multivariate Cox analysis to identify the independent prognostic factors of OS and CSS. All statistical analyses in this study were performed with R software (version 4.1.3; https://www.r-project.org/), and a two-tailed P <0.05 was considered statistically significant.

## Results

### Baseline characteristics

In this study, a total of 5,313 patients diagnosed with microscopically confirmed gastric cancer and liver metastasis between 2010 and 2015 were extracted from the SEER database. Based on the inclusion and exclusion criteria in our study, 3,001 patients were enrolled for further analysis (**Figure 1**). Of those, 328 (12.3%) patients underwent PTR, whereas 2,673 (87.7%) did not. The baseline characteristics of the overall population are shown in **Table 1**. A 1:1 PSM analysis was performed to balance the available covariates such as sex, race, degree of tumor differentiation, tumor T-stage, tumor N-stage, pathological type, tumor location, and tumor size between the PTR and no-PTR groups, a 1:1 PSM analysis was performed. The baseline characteristics that were well-balanced in the matched cohort after PSM are also summarized in **Table 1**.

**Table 1:** Baseline characteristics of the selected patients before and after propensity score matching.

Variables	Before PSM			After PSM		
	no-PTR	PTR	P-value	no-PTR	PTR	P-value
	n = 2,673 (%)	n = 328 (%)		n = 215 (%)	n = 215 (%)	
**Year**			0.150			0.305
2010	406 (15.2)	61 (18.6)		29 (13.5)	42 (19.5)	
2011	426 (15.9)	60 (18.3)		27 (12.6)	35 (16.3)	
2012	416 (15.6)	59 (18.0)		38 (17.7)	38 (17.7)	
2013	475 (17.8)	51 (15.5)		36 (16.7)	34 (15.8)	
2014	469 (17.5)	49 (14.9)		44 (20.5)	32 (14.9)	
2015	481 (18.0)	48 (14.6)		41 (19.1)	34 (15.8)	
**Age**			0.817			0.772
<65	1,245 (46.6)	150 (45.7)		101 (47.0)	105 (48.8)	
>65	1,428 (53.4)	178 (54.3)		114 (53.0)	110 (51.2)	
**Sex**			<0.001			0.919
Female	761 (28.5)	123 (37.5)		72 (33.5)	74 (34.4)	
Male	1,912 (71.5)	205 (62.5)		143 (66.5)	141 (65.6)	
**Race**			<0.001			0.770
Black	419 (15.7)	73 (22.3)		41 (19.1)	47 (21.9)	
White	1,922 (71.9)	195 (59.5)		137 (63.7)	133 (61.9)	
Other	332 (12.4)	60 (18.3)		37 (17.2)	35 (16.3)	
**Marital**			0.284			0.139
Divorced	235 (8.8)	25 (7.6)		23 (10.7)	13 (6.0)	
Married	1,514 (56.6)	204 (62.2)		122 (56.7)	143 (66.5)	
Single	456 (17.1)	47 (14.3)		30 (14.0)	27 (12.6)	
Other	468 (17.5)	52 (15.9)		40 (18.6)	32 (14.9)	
**Income**			0.716			0.922
<60,000	1,141 (42.7)	136 (41.5)		88 (40.9)	86 (40.0)	
>60,000	1,532 (57.3)	192 (58.5)		127 (59.1)	129 (60.0)	
**Residence**			0.426			0.793
Metropolitan	2,403 (89.9)	301 (91.8)		198 (92.1)	194 (90.2)	
Rural	118 (4.4)	14 (4.3)		8 (3.7)	10 (4.7)	
Urban	152 (5.7)	13 (4.0)		9 (4.2)	11 (5.1)	
**Source**			0.053			0.501
Hospital	2,595 (97.1)	325 (99.1)		209 (97.2)	212 (98.6)	
no-Hospital	78 (2.9)	3 (0.9)		6 (2.8)	3 (1.4)	
**PRCDA**			0.295			0.086
No	788 (29.5)	87 (26.5)		69 (32.1)	52 (24.2)	
Yes	1,885 (70.5)	241 (73.5)		146 (67.9)	163 (75.8)	
**Original**			0.813			0.795
Hispanic	2,165 (81.0)	268 (81.7)		181 (84.2)	178 (82.8)	
Non-Hispanic	508 (19.0)	60 (18.3)		34 (15.8)	37 (17.2)	
**Grade**			<0.001			0.944
Grade I	61 (2.3)	14 (4.3)		7 (3.3)	6 (2.8)	
Grade II	649 (24.3)	90 (27.4)		57 (26.5)	55 (25.6)	
Grade III	1,268 (47.4)	156 (47.6)		93 (43.3)	99 (46.0)	
Grade IV	56 (2.1)	18 (5.5)		10 (4.7)	12 (5.6)	
Unknown	639 (23.9)	50 (15.2)		48 (22.3)	43 (20.0)	
**T**			<0.001			0.874
T0	10 (0.4)	0 (0)		0 (0)	0 (0)	
T1	541 (20.2)	24 (7.3)		28 (13.0)	23 (10.7)	
T2	90 (3.4)	18 (5.5)		12 (5.6)	16 (7.4)	
T3	265 (9.9)	125 (38.1)		81 (37.7)	78 (36.3)	
T4	401 (15.0)	139 (42.4)		72 (33.5)	76 (35.3)	
TX	1,366 (51.1)	22 (6.7)		22 (10.2)	22 (10.2)	
**N**			<0.001			0.314
N0	1,019 (38.1)	110 (33.5)		89 (41.4)	94 (43.7)	
N1	989 (37.0)	80 (24.4)		65 (30.2)	73 (34.0)	
N2	84 (3.1)	52 (15.9)		25 (11.6)	27 (12.6)	
N3	52 (1.9)	78 (23.8)		24 (11.2)	13 (6.0)	
NX	529 (19.8)	8 (2.4)		12 (5.6)	8 (3.7)	
**Histology**			<0.001			0.713
adenocarcinoma	1,797 (67.2)	136 (41.5)		107 (49.8)	101 (47.0)	
Gastrointestinal stromal	118 (4.4)	55 (16.8)		42 (19.5)	37 (17.2)	
Intestinal type	186 (7.0)	51 (15.5)		25 (11.6)	24 (11.2)	
Signet ring cell	165 (6.2)	18 (5.5)		9 (4.2)	13 (6.0)	
Other	407 (15.2)	68 (20.7)		32 (14.9)	40 (18.6)	
**Tumor location**			<0.001			0.753
Body	228 (8.5)	38 (11.6)		22 (10.2)	25 (11.6)	
Cardia	1,042 (39.0)	49 (14.9)		53 (24.7)	46 (21.4)	
Fundus	142 (5.3)	17 (5.2)		9 (4.2)	11 (5.1)	
Gastric antrum	333 (12.5)	94 (28.7)		45 (20.9)	44 (20.5)	
Greater	93 (3.5)	18 (5.5)		10 (4.7)	15 (7.0)	
Lesser	160 (6.0)	22 (6.7)		7 (3.3)	12 (5.6)	
Pylorus	44 (1.6)	11 (3.4)		5 (2.3)	7 (3.3)	
Other	631 (23.6)	79 (24.1)		64 (29.8)	55 (25.6)	
**Tumor size**			<0.001			0.408
<1 cm	1,004 (37.6)	229 (69.8)		124 (57.7)	135 (62.8)	
>1 cm	130 (4.9)	49 (14.9)		30 (14.0)	31 (14.4)	
Unknown	1,539 (57.6)	50 (15.2)		61 (28.4)	49 (22.8)	
**Chemotherapy**			0.234			0.425
No	1,150 (43.0)	153 (46.6)		76 (35.3)	85 (39.5)	
Yes	1,523 (57.0)	175 (53.4)		139 (64.7)	130 (60.5)	
**Radiotherapy**			0.124			0.888
No	2,328 (87.1)	296 (90.2)		185 (86.0)	187 (87.0)	
Yes	345 (12.9)	32 (9.8)		30 (14.0)	28 (13.0)	

### Survival outcomes of PTR before matching

**Figure 2:**
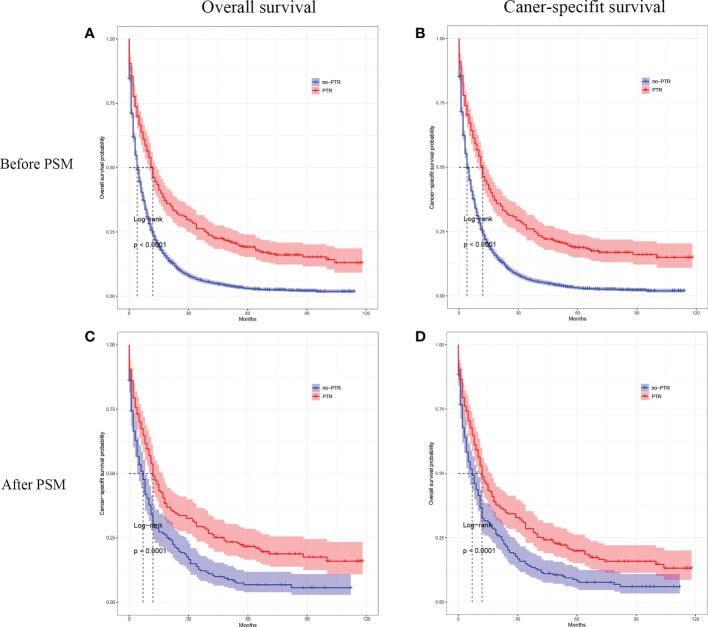
Kaplan–Meier curves for overall survival and cancer-specific survival of patients with primary tumor resection versus without primary tumor resection before and after the propensity score matching.

A Kaplan‐Meier analysis was performed to calculate the OS and CSS of the overall population cohort before PSM. The results showed that patients with PTR had a significantly higher OS and CSS rate than those without PTR (log-rank p <0.001, **Figure 2A**; log-rank p <0.001, **Figure 2B**). The median OS was 12.0 months (95% CI, 10 months to 14 months) for those who underwent PTR and 4 months (95% CI, 4 months to 5 months) for those without PTR, respectively. The median CSS for those who underwent PTR was 12.0 months (95% CI, 10 months to 14 months) and 4 months (95% CI, 4 months to 5 months) for those without PTR, respectively.

### Survival outcomes of PTR after matching

In the matched cohort, patients with PTR also had a significantly higher OS and CSS rate than those without PTR (log-rank p <0.001, **Figure 2C**; log-rank p <0.001, **Figure 2D**). After PMS, the median OS was 12.0 months (95% CI, 10 months to 17 months) for those who underwent PTR and 7 months (95% CI, 5 months to 10 months) for those without PTR, respectively. The median CSS for those who underwent PTR was 12.0 months (95% CI, 11 months to 17 months) and 7 months (95% CI, 5 months to 8 months) for those without PTR, respectively. Furthermore, the Cox proportional hazards model was performed to confirm the prognostic significance of PTR in patients with gastric cancer and liver metastasis (**Table 2**). In the univariable Cox analysis for the matched cohort, PTR, age, degree of tumor differentiation, tumor N-stage, pathological type, tumor size, and chemotherapy were significantly associated with OS, and these variables were all included in the multivariate Cox analysis. Multivariate Cox analysis revealed that PTR, age, degree of tumor differentiation, and chemotherapy were independent prognostic factors for OS of the patients with gastric cancer and liver metastasis. Specifically, PTR was a significant protective factor for OS (HR: 0.427; 95% CI, 0.325 to 0.561, P <0.001). As for mortality risk, it was higher in patients with age >65 y, poorly differentiated or undifferentiated and no chemotherapy than in patients with age <65 y, well-differentiated or moderately differentiated and chemotherapy, respectively (**Table 2**).

**Table 2:** Univariate and multivariate analysis of prognostic factors for OS in the propensity score matched cohort.

Variables	Univariable			Multivariable		
	HR	95%CI	P-value	HR	95%CI	P-value
**PTR**						
No	reference			reference		
Yes	0.666	0.542 to 0.817	<0.001	0.427	0.325 to 0.561	<0.001
**Year**						
2010	reference					
2011	1.2658	0.877 to 1.826	0.208			
2012	1.2055	0.848 to 1.713	0.297			
2013	1.3449	0.937 to 1.930	0.108			
2014	0.9502	0.662 to 1.363	0.782			
2015	1.3038	0.909 to 1.869	0.149			
**Age**						
<65	reference			reference		
>65	1.646	1.341 to 2.020	<0.001	1.421	1.081 to 1.869	0.012
**Sex**						
Female	reference					
Male	0.9822	0.792 to 1.218	0.870			
**Race**						
Black	reference					
White	1.362	1.049 to 1.770	0.021			
Other	1.298	0.926 to 1.820	0.131			
**Marital**						
Divorced	reference					
Single	235 (8.7)	0.472 to 1.146	0.174			
Married	0.7286	0.506to 1.050	0.090			
Other	0.9861	0.648to 1.500	0.948			
**Income**						
<60 000	reference					
>60 000	1.07	0.868 to 1.319	0.526			
**Residence**						
Metropolitan	reference					
Rural	1.5411	0.946 to 2.510	0.082			
Urban	0.9137	0.561 to 1.487	0.717			
**Source**						
Hospital	reference					
no-Hospital	1.101	0.546 to 2.219	0.788			
**PRCDA**						
No	reference					
Yes	0.9028	0.719 to 1.132	0.377			
**Original**						
Hispanic	reference					
Non-Hispanic	1.312	0.994 to 1.733	0.055			
**Grade**						
Grade I	reference			reference		
Grade II	2.246	1.134 to 4.450	0.020	2.288	1.029 to 5.086	0.042
Grade III	2.707	1.381 to 5.306	0.004	3.552	1.604 to 7.866	0.002
Grade IV	1.467	0.659 to 3.269	0.348	5.298	2.021to13.891	<0.001
**T**						
T4	reference					
T3	0.9059	0.711 to 1.155	0.425			
T2	0.7536	0.487 to 1.167	0.205			
T1	1.2672	0.904 to 1.776	0.169			
**N**						
N0	reference			reference		
N1	1.710	1.320 to 2.215	<0.001	1.113	0.792 to 1.565	0.538
N2	2.109	1.506 to 2.955	<0.001	1.345	0.870 to 2.076	0.182
N3	1.659	1.120 to 2.459	0.012	1.013	0.605 to 1.695	0.961
**Histology**						
adenocarcinoma	reference			reference		
Signet ring cell	0.8855	0.551 to 1.424	0.616	0.824	0.391 to 1.738	0.611
Gastrointestinal stromal	0.2453	0.173 to 0.348	<0.001	0.256	0.143 to 0.460	<0.001
Intestinal type	0.8391	0.600 to 1.173	0.305	1.343	0.893 to 2.019	0.157
Other	0.8693	0.642 to 1.177	0.365	1.286	0.897 to 1.842	0.171
**Tumor location**						
Body	reference					
Cardia	1.3764	0.922 to 2.055	0.118			
Fundus	0.6846	0.367 to 1.276	0.233			
Gastric antrum	1.2946	0.859 to 1.952	0.218			
Greater	1.0345	0.589 to 1.814	0.906			
Lesser	1.1936	0.659 to 2.164	0.560			
Pylorus	1.4554	0.737 to 2.875	0.280			
Other	1.1605	0.779 to 1.729	0.464			
**Tumor size**						
<1 cm	reference			reference		
>1 cm	0.5968	0.429 to 0.829	0.002	1.271	0.870 to 1.857	0.214
**Chemotherapy**						
No	reference			reference		
Yes	0.4072	0.325 to 0.510	<0.001	0.407	0.304 to 0.544	<0.001
**Radiotherapy**						
No	reference					
Yes	1.237	0.923 to 1.658	0.154			

### Survival outcomes stratified by age, tumor differentiation, and chemotherapy

**Figure 3:**
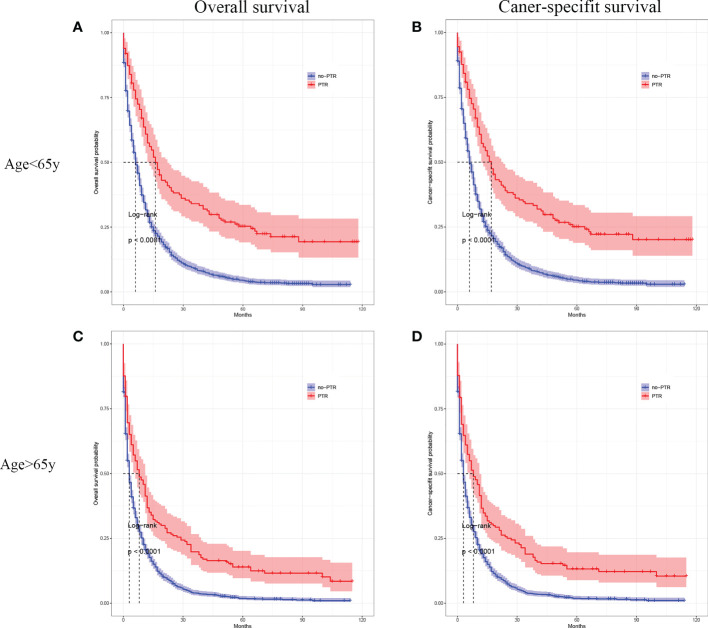
Kaplan–Meier curves for overall survival and carcinoma-specific survival of patients with primary tumor resection versus without primary tumor resection stratified by age in the unmatched cohort.

**Figure 4:**
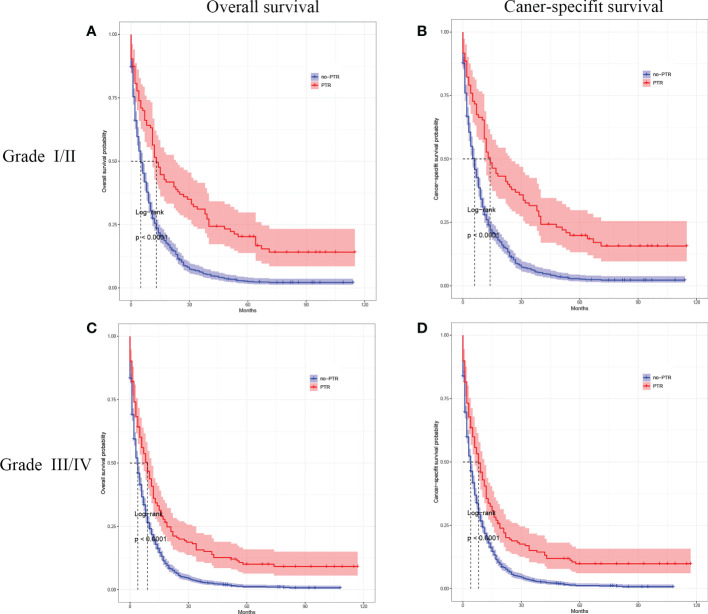
Kaplan–Meier curves for overall survival and carcinoma-specific survival of patients with primary tumor resection versus without primary tumor resection stratified by tumor differentiation in the unmatched cohort.

**Figure 5:**
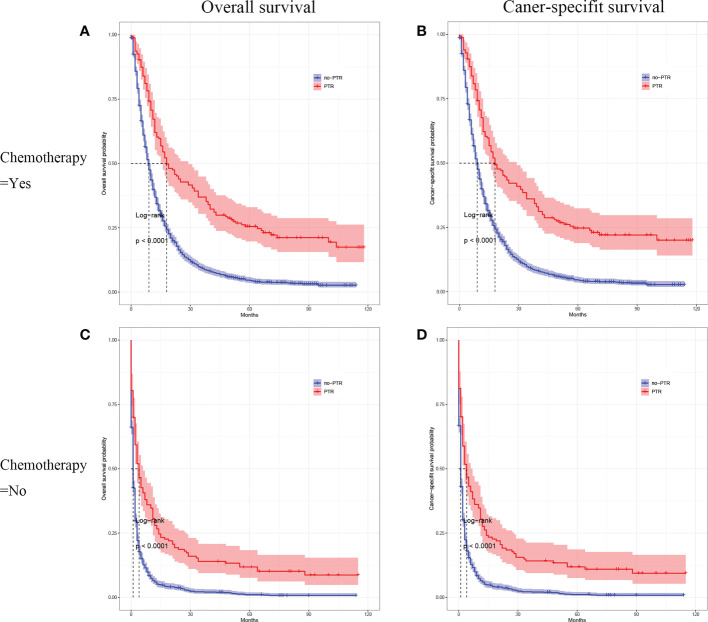
Kaplan–Meier curves for overall survival and carcinoma-specific survival of patients with primary tumor resection versus without primary tumor resection stratified by chemotherapy in the unmatched cohort.

Cox analysis showed that PTR, age, tumor differentiation, and chemotherapy were independent prognostic factors for OS in patients with gastric cancer and liver metastasis. Therefore, we used subgroup analysis to explore the effect of PTR on overall survival. In the age subgroup with a median age of <65, the overall survival and cancer-specific survival of the PTR group were longer than the no-PRT group (log-rank P <0.001, **Figure 3A** and log-rank P <0.001, **Figure 3B**). Similarly, in the age subgroup with a median age of >65, the overall survival and cancer-specific survival of the PTR group were longer than the no-PRT group (log-rank P <0.001, **Figure 3C** and log-rank P <0.001, **Figure 3D**). Based on subgroup analysis of tumor differentiation, overall survival and cancer-specific survival rate of the PTR group were significantly higher than those of the no-PTR group (log-rank P <0.001, **Figure 4A** and log-rank P <0.001, **Figure 4B**) in patients with well or moderately differentiated tumors (Grades I–II). Also, in the patients with poorly differentiated or undifferentiated tumors (Grades III–IV), the overall survival and cancer-specific survival rates of the PTR group were significantly higher than those of the no-PTR group (log-rank P <0.001, **Figure 4C** and log-rank P <0.001, **Figure 4D**). In the chemotherapy subgroup, the overall survival and cancer-specific survival of the PTR group were longer than the no-PRT group in the patients with chemotherapy (log-rank P <0.001, **Figure 5A** and log-rank P <0.001, **Figure 5B**). Likewise, in the subgroup without chemotherapy, the overall survival and cancer-specific survival of the PTR group were also longer than the no-PRT group (log-rank P <0.001, **Figure 5C** and log-rank P <0.001, **Figure 5D**).

## Discussion

Gastric cancer with liver metastasis (GCLM), the deadliest disease, is considered an advanced gastric cancer with a poor prognosis (10). GCLM includes two types: synchronous metastases and metachronous metastases. Synchronous metastases are defined as metastases that appear before surgical resection or after gastrectomy within 6 months, whereas metachronous metastases occur after gastrectomy over 180 days (11–13). At the initial time of diagnosis, about 4%–14% of gastric cancer patients presented with liver metastases due to its non-specific symptoms (14). However, approximately 37% of gastric cancer patients developed metachronous liver metastases after gastrectomy (15). GCLM was considered an incurable disease and the first-line treatment for it was systematic chemotherapy according to EMSO’s or NCCN’s guidelines (16, 17). The role of surgical resection is still controversial and debated because of limited data to support routine surgery resection (18). A gastrectomy is considered to be performed when severe gastrointestinal symptoms such as obstruction or refractory hemorrhage occur. However, more and more surgeons investigated the role of surgery resection in patients with liver metastases inspired by survival benefits of surgery of colorectal cancer patients with liver metastases (13, 19, 20). Some retrospective studies showed that surgery, including liver resection or primary tumor resection in GCLM, significantly improved survival (11, 21–23). But owing to the single center and small case data, the benefit of primary tumor resection for gastric patients with liver metastases needs to be further confirmed.

In our study, all 328 patients from multiple centers underwent gastric primary tumor resection, including partial, sub-total, or total gastrectomy, which confirmed that primary tumor resection, as an independent protective factor, improves overall and cancer-specific survival of gastric cancer patients with liver metastasis. Firstly, using the data collected from the SEER database, this study showed big improvements in survival outcomes of PTR to GCLM patients in the overall patient cohort. Secondly, after PSM with the aim of balancing the potential covariates between the primary tumor resection and non-surgery cohort, the better OS and CSS of GCLM patients in the PTR group than in the no-PTR group, and our research confirmed that PTR, age, degree of tumor differentiation, and chemotherapy were independent prognostic factors for OS of the patients with gastric cancer and liver metastasis. Specifically, PTR was a significant protective factor for OS (HR: 0.427; 95% CI, 0.325 to 0.561, P <0.001). Thirdly, subgroup analysis showed that GCLM patients with age <65 y, well-differentiated or moderately differentiated, and chemotherapy have better overall survival in the PTR group than the no-PRT group.

However, a recent clinical trial showed contradictory and inconsistent results of gastrectomy in GCLM patients. The AIO-FLOT3 trial showed that patients who were treated with gastrectomy and chemotherapy had better OS (22.9 vs. 10.7 months) than those treated with chemotherapy alone, while the REGATTA trial reported that the overall survival (OS) and progression-free survival (PFS) of patients who were treated with palliative surgery plus chemotherapy had no significant difference compared to those who were treated with chemotherapy alone. But in other metastatic diseases such as ovarian and renal tumors, primary tumor resection can result in better survival, as reported in clinical trials (24–26). Why primary tumor resection can prolong the survival of metastatic tumor patients remains uncertain. Recently, a possible mechanism was reported that recovering the immune system by primary tumor resection improved patient survival (27, 28). Also, another possible mechanism was reported that the high circulating tumor cells (CTCs) in the blood resulted from primary tumor causes such as liver or lung metastases (29, 30). So, primary tumor resection might prolong the survival of metastatic tumor patients by reducing circulating tumor cells (27). Therefore, we consider that primary tumor resection improves the survival of gastric cancer patients with liver metastasis possibly by recovering the immune system and reducing circulating tumor cells.

Some opponents argue that resection of the primary tumor in metastasis tumor patients might delay the start of systemic therapy and increase postoperative complications, thus affecting survival time (31). Besides, medical costs and the quality of postoperative life should be considered before surgery (32). Consequently, a multidisciplinary team should evaluate whether gastric cancer patients with liver metastasis are suitable for excising the primary tumor.

There are several limitations to this study. Firstly, this study based on the SEER database has the natural limitation of incomplete information. This database didn’t record patients’ performance status and comorbidities; preoperative or postoperative complications; the details of liver metastases and their treatment; or chemotherapy regimens. Those factors affect the prognosis of patients with GCLM, but we cannot acquire that relevant information from the SEER database. Secondly, due to its retrospective nature, selection bias in our study was unavoidable. So we used PSM analysis to balance covariates between the PTR and no-PTR groups to reduce other confounding biases. However, there might be other unobserved confounders not included in the propensity score matching. Thirdly, it is not reported whether the clinical symptoms of the primary gastric tumor might affect the selection of PTR surgery. All in all, only a well-designed randomized control trial can avoid those biases and verify our findings.

## Conclusions

In conclusion, this propensity score-matched, population-based study showed that primary tumor resection yields an association with favorable overall and cancer-specific survival in gastric cancer patients with liver metastasis. A well-designed prospective randomized controlled trial should be performed to confirm this initial conclusion.

## Data availability statement

The datasets presented in this study can be found in online repositories. The names of the repository/repositories and accession number(s) can be found in the article/supplementary material.

## Author contributions

Conception and design: YW and GL. Administrative support: YW. Provision of study materials or patients: JW, HZ, and CZ. Collection and assembly of data: JY, YL, ZM, and ZL. Data analysis: JW. Manuscript writing: All authors. All authors listed have made a substantial, direct, and intellectual contribution to the work and approved it for publication.

## Funding

This study was supported by the National Natural Science Foundation of China (No. 8180102403 ; No. 30671987) and National Key Clinical Discipline.

## Conflict of interest

The authors declare that the research was conducted in the absence of any commercial or financial relationships that could be construed as a potential conflict of interest.

## Publisher’s note

All claims expressed in this article are solely those of the authors and do not necessarily represent those of their affiliated organizations, or those of the publisher, the editors and the reviewers. Any product that may be evaluated in this article, or claim that may be made by its manufacturer, is not guaranteed or endorsed by the publisher.
